# Multimodality in language education: implications of a multimodal affective perspective in foreign language teaching

**DOI:** 10.3389/fpsyg.2023.1283625

**Published:** 2023-10-09

**Authors:** Xiaoshuang Guo

**Affiliations:** School of Foreign Studies, China University of Political Science and Law, Beijing, China

**Keywords:** academic writing, language teaching, emotion, multimodal, GFL

## Abstract

Foreign language learners often encounter various emotional challenges within academic environments, which can hinder their progress in developing literacy skills. Effective language instruction should encompass teaching approaches that acknowledge the emotional requirements of students. To address this need, we propose a multimodal affective methodology designed to evaluate emotions in foreign language education scenarios. This methodology also holds the potential to elucidate the pedagogic contributions of various emotional variables to academic outcomes. Our study focuses on German as a foreign language (GFL) learning and utilizes it as an example to investigate ways to improve writing proficiency. The study explores the effects of integrating multimodal corrective feedback (MCF) into academic writing exercises. We delve into suitable modalities for analyzing emotions in academic writing practices. Furthermore, we investigate how the choice of corrective feedback mode intricately influences the nature of feedback itself and subsequently influences students’ emotional responses. Through a comprehensive exploration of the interplay between distinct modes of delivering feedback and their impacts on learners’ engagement, this investigation strives to decode the intricate dynamics of emotions that underlie language acquisition. With these insights, the study discusses how teachers can enhance their teaching strategies by combining changes in learners’ emotional states and providing emotional support.

## 1. Introduction

Emotions play a critical role in daily tasks by influencing cognitive capacity and providing the necessary energy for behavioral action (Ekman, 1992; Liu, 2022). In this turn, there exists a direct link between emotions and language learning. Negative emotions are often a source of challenges for foreign language learners in academic writing curriculums. Despite the substantial amount of research conducted on the factors affecting writing processes and products, the role of emotion has been given scant attention in writing studies. This study aims to evaluate and determine the appropriateness of various modalities for analyzing emotions within the German as a Foreign Language (GFL) learning context. We thus introduce a multimodal method to accurately assess emotions in academic writing classes.

Considering students’ emotions in their academic literacy development can provide insight into the conditions under which emotions are associated with academic functioning. In recent years, researchers in positive psychology have recognized the effectiveness of positive reinforcement in promoting efficient learning among students (Dewaele et al., 2019; Li, 2020). Similarly, negative emotions are often observed to cause disruptions in students, but they can also stimulate activation in long-term memory associated with learning. Most research on emotions and academic competence has focused on negative emotions such as anger (Gan et al., 2022), anxiety (Thompson and Lee, 2013), and boredom (Shen, 2022) with minimal attention paid to other positive emotions. Influenced by positive psychology scholars, who realized that emotion is one of the most effective ways to enhance student’s learning efficiency (Li, 2020; Ergün and Dewaele, 2021; Yang et al., 2021). Researchers advocate a shift in the focus from repairing negative affection to fostering positive subjective experiences that promote hope, courage, and happiness (Dewaele et al., 2019; Han and Wang, 2021).

Emotions are complex and expressed through various modalities, including facial expressions, gestures, and oral expressions (Liu P. et al., 2022). Scholars agree that emotions consist of multiple components and are thus complex to study (Gao and Cui, 2022; Liu et al., 2023; Wen et al., 2023). Several studies claim that a single modality is insufficient for providing a comprehensive understanding of emotions due to their complexity (Goncalves et al., 2017; Liu W. et al., 2022). To address this issue, this study focuses on emotions and their interplay in a multimodal affective perspective.

This paper delves into the potential of a multimodal affective approach to enrich language education, particularly its pertinence within the context of German as a foreign language (GFL) of academic Writing curriculum. In the realm of written production assessment, educators encounter a spectrum of choices in delivering corrective feedback to students. Normally, educators deliver corrective feedback through the written modality. Instead, the paradigm shift toward digital and distance learning has accentuated the necessity for enhanced pedagogical training beyond the written modality (Trigo et al., 2019; Ibáñez et al., 2022). The incorporation of technology has expanded the scope through which educators can provide multimodal corrective feedback (MCF) to their students. Rather than confining themselves to conventional textual output, educators now possess the capacity to provide MCF through video channels. Nevertheless, instructors must exercise awareness of how modalities might impact both the nature of their feedback and the emotional responses of students. By harnessing an array of nonverbal cues encompassing gestures, eye contact, and facial expressions, this modality becomes a potent tool to captivate learners’ sensory engagement. This work posits an imperative shift, acknowledging the multimodal nature of emotions and their constructive role in foreign language learning.

## 2. Literature review

The expression and identification of emotions by individuals are recognized to be multifaceted. The implementation of a multimodal affective perspective offers the potential to augment our knowledge of the interplay between academic functioning and emotions, as it enables chances for new research directions. We conduct a review of current emotional studies within academic contexts.

### 2.1. Multimodal emotion and multiliteracies

Learning is emotional and cognitive, as affective states cause or are accompanied by changes in how individuals process information (Dylman and Bjarta, 2019). The learning environment is multimodal, wherein students engage with instructors (Forceville, 2005; Liu, 2022). Teachers express their emotions through various modalities (Unsworth, 2001; Kress and Van Leeuwen, 2020). The concept of multimodality originates from systemic functional linguistics, a theory developed by Michael Halliday and his colleagues (Halliday and Matthiessen, 2013). Multimodality involves various aspects of interaction and environments, including speech and nonverbal elements such as visual, aural, embodied, and spatial cues (Jewitt, 2013). Generally, multimodality involves the use of two or more modalities.

Different from the disparate perspective, researchers have recently advocated emotion as highly integrated, multilevel, and complex systems (Rothermund and Koole, 2018; Scherer and Moors, 2019). Recent studies have revealed that multimodal data can enhance accuracy and provide better insight into emotions and academic literacy development process (Soleymani et al., 2012; Lee and Anderson, 2017), while single-method assessments are often problematic in monitoring dynamic students’ emotional states (Chrysafiadi and Virvou, 2013).

Some scholars focused on modality-specified features, such as language resources (Kövecses, 2003), facial expressions (Ekman and Friesen, 1978), vocal features (Scherer, 2003), psychological reactions (Fan et al., 2016; Dzedzickis et al., 2020; Yang, 2021) or body language (Behoora and Tucker, 2015). To enhance the reliability of obtained results, a multimodal affective perspective is needed, as most studies of emotion analysis have been modality-specific. With the rapid development of machine learning and affective computing (Scherer and Moors, 2019; Liu et al., 2023), automated emotion evaluation is a potential trend. Those studies offer helpful lessons on assessing multimodal emotions, as the experimental results indicate that multimodal systems attain better effects on performance than unimodal counterparts (Goncalves et al., 2017).

Although emotion analysis has been extensively investigated, existing studies have not focused on designing a multimodal affective framework. There is an urgent need for an ecologically valid instrument for measuring emotions in academic writing practice. To this end, we provide a multimodal affective framework to analyze the complex multimodal meaning of emotions.

### 2.2. Emotions and foreign language learning

Emotions and language learning are interconnected (Dewaele and MacIntyre, 2014; Liu, 2022). Scientists have noted that emotions serve as a crucial component of individuals, influencing their learning behavior, cognitive capacity, and interpersonal relationships (Ekman, 1992). In response to this recognition, educational theorists and practitioners have begun to emphasize the significant value of integrating positive emotional engagements into curricula. This emphasis stems from the understanding that doing so can substantially enhance students’ engagement in the learning process and foster more positive attitudes toward their educational experiences (Vicente-Yagüe Jara, 2018).

Moreover, the growing body of research has delved into the effects of affectivity on various dimensions of well-being, emotional intelligence, and academic accomplishments (Sansuán, 2020; López Martínez et al., 2023). For instance, Sansuán (2020) examined the consequences of emotional engagement within the context of poetry education among adolescents, shedding light on the profound impact that emotions can have on the learning process. Collectively, these investigations support the idea that emotions should not be viewed as mere byproducts of the educational process. Instead, they should be regarded as integral and influential factors that have the potential to significantly shape the educational experiences of both children and adolescents.

To reveal the interplay between emotions and the language learning process, researchers have explored various emotional states in the foreign language classroom. Negative emotions such as anxiety (Thompson and Lee, 2013), anger (Gan et al., 2022), and boredom (Shen, 2022) or positive emotions like enjoyment (Pavelescu and Petric, 2018; Yang, 2021) are important for successful learning (Burić et al., 2016). While negative emotions narrow the mindset, positive emotions stimulate innovative ideas. Some researchers constructively move positive psychology forward by putting negative and positive realms together (Ryff, 2022). Literature has examined that emotion impacts learning in the foreign language classroom (Han and Wang, 2021; Wang and Ye, 2021). Learning is emotional and cognitive, as affective states cause or are accompanied by changes in how individuals process information (Dylman and Bjarta, 2019), student with high language anxiety can positively contribute to seeking assistance for taking actions in learning engagement (Ryff, 2022).

As we delve into the analysis of emotions in foreign language learning, specifically focusing on the implementation of corrective feedback (CF). Existing literature underscores the profound impact of CF in foreign language learning contexts (Langum and Sullivan, 2017; Han and Hyland, 2019; Yu et al., 2020). CF facilitates students in the rectification of misspellings, the avoidance of grammatical errors, and the refinement of language accuracy. However, the emotional dimensions associated with corrections remain relatively unexplored.

While written corrective feedback (WCF) is mostly employed to revise students’ errors due to their convenience and validity, there is a dearth of research that delves into the emotional factors within the context of the multimodal approach (Scherer and Moors, 2019). With this research gap in mind, we aim to provide a multimodal affective perspective on the emotional processes and components of multimodal emotional expressions, which intends to bring new perspectives to the attention of emotion in academic settings, especially investigating the underlying mechanisms in foreign language writing lectures. This study analyzes the inherent multimodality embedded within the construct of teacher corrective feedback, which holds the potential to catalyze fostering positive emotional experiences, such as a sense of enjoyment.

## 3. A bibliometric analysis of emotion in academic contexts

In our pursuit of the intricate involvement of emotions in the development of academic literacy, we conducted a thorough bibliometric analysis of existing research to identify prevalent clusters and potential connections. To collect relevant data, we employed the Web of Science search engines, utilizing the keywords “Emot*, academic Lit*.” These keywords were selected to explore how emotions and academic literacy concepts have been integrated into existing emotion research literature.

**Figure 1:**
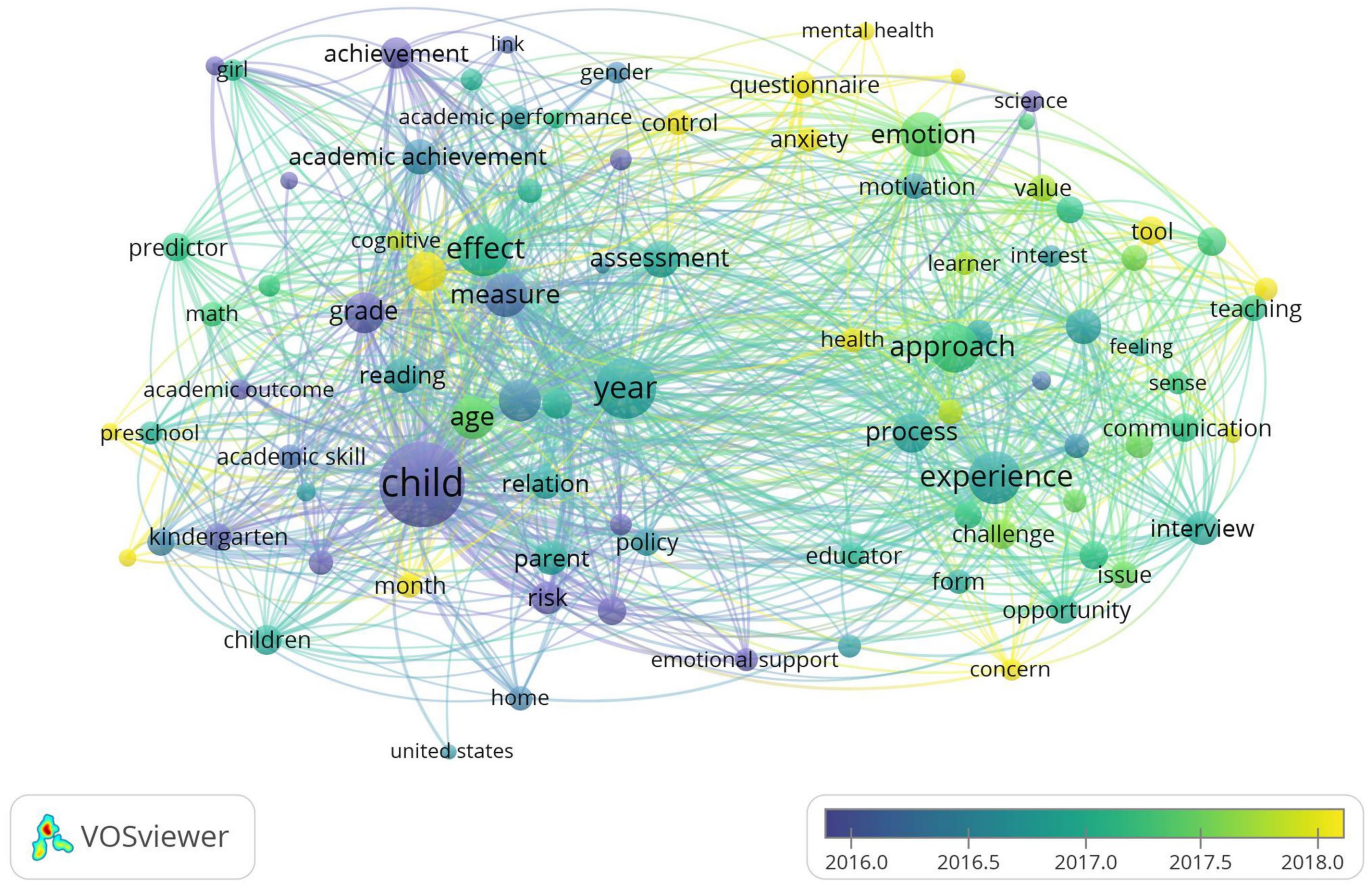
Bibliometric analysis with keywords “Emot*, academic Lit*” in VOS Viewer.

Our search yielded 676 results in total, with the documents published between 1995 and 2023. Figure 1 visualizes the results and their interconnections, which offers a comprehensive view of the landscape of emotion research within the realm of academic literacy. It has brought to light the recurring themes of “approach,” “measure,” and “process” as commonly referenced concepts when evaluating emotions within academic contexts. Notably, self-reported questionnaires emerge as the predominant method employed for this purpose (Scherer and Moors, 2019). While substantial progress has been made in understanding the impact of emotions on foreign language learning, there remains a gap in research about using a multimodal perspective for analyzing emotions. The proposed multimodal affective approach has the potential to analyze diverse educational settings using a unified and applicable paradigm for assessing emotional factors. Given the relative shortage of attention in previous research, this method could offer a more comprehensive understanding of the issue.

Given the profound impact of emotions on students’ learning activities, it becomes imperative to cultivate positive emotions within the learning process. Emotions serve a functional purpose by equipping individuals with the capacity to respond effectively to challenging educational situations. Emotions are not limited to verbal expressions or self-reporting. They manifest through various modalities, including nonverbal cues. It is essential to note that nonverbal behaviors, encompassing diverse modes such as gestures, posture, gaze, and movements, constitute an integral facet of teachers’ CF and should not be overlooked when assessing the pedagogical effectiveness of their feedback.

Table 1 enumerates various modalities in the multimodal affective approach. Multimodality encompasses various facets such as facial expressions, body movements, vocal intonations, and the utilization of technological tools, all of which accompany teachers’ corrective feedback. This paradigm can analyze different modalities of emotions by using the equally applicable paradigm so that the findings can be simultaneously compared. The integration of multiple data streams holds the potential to enhance the precision of emotion recognition, as evidenced in recent studies (Liu et al., 2023; Wen et al., 2023). These methods offer a relatively straightforward means of data collection, often involving video frame analysis. Within this visual record of the teacher’s multimodal corrective feedback, both verbal and nonverbal semiotic elements, including gestures, gazes, and postures, have been meticulously captured (Table 2).

**Table 1:** Modalities in the multimodal affective approach.

Modality	Sensors or tools	Description	Strength	Weakness
Auditory modality	Speech recognition tools	The use of tone, intonation and rhythm	Covey emotions and highlight key point; capable for assessing spontaneous emotion	Lack of theoretical framework in emotion detection
Visual modality	Image recognition	Visual aids, such as diagrams and images	Simplify complex concepts; engage learners with visual information	Limiting usability and accessibility; Variability of human emotional expressions
Gestural modality	Facial Action Coding System (FACS)	Assessing facial motion and gestures	center of emotion detection; reinforce verbal explanations; Easily detectable in and out of classroom	Variability of human emotional expressions, limited success rate for sole use, lacks detailed temporal information
Kinesthetic modality	Visual gesture recognition system	Assessing body movement	Indicator of emotions; indicator to interactive experiences; reinforce emotional connections; less obtrusive methods	Lack of capability to detect fine body movements with emotions; require more resources and time
Text modality	NLP Tools and questioner	Written content	Easily gathered data; provide detailed explanations and directly connected to emotional expressions; suitable for various contexts	Not real-time; data need post-processed; might lead to cognitive overload

**Table 2:** Distribution of students’ emotional reactions to MCF/WCF according to the questionnaire.

Questions	Strongly disagree	Disagree	Neutral	Agree	Strongly agree
Section 1: Multimodal corrective feedback and intellectual engagement					
(1) I believe that teacher’s multimodal corrective feedback is beneficial to improve my English.	0	1 (3.33%)	3 (10%)	3 (10%)	23 (76.67%)
(2) Multimodal corrective feedback plays a significant role in helping me recognize and correct my mistakes in writing.	1 (3.33%)	0	5 (16.67%)	18 (60%)	7 (23.33%)
(3) The use of multimodal corrective feedback attracts my attention in the revision process.	2 (6.67%)	0	1 (3.33%)	6 (20%)	21 (70%)
Section 2: Multimodal corrective feedback and emotional response					
(4) The teacher’s use of multimodal corrective feedback made me feel more confident about my learning progress.	0	2 (6.67%)	4 (13.33%)	11 (36.67%)	13 (43.33%)
(5) I find the multimodal corrective feedback enjoyable.	0	1 (3.33%)	3 (10%)	4 (13.33%)	22 (73.33&)
(6) I find Teacher’s written corrective feedback enjoyable.	4 (13.33%)	3 (10%)	14 (46.67%)	7 (23.33%)	2 (6.67%)
Section 3: Preference of different feedback modes					
(7) The combination of visual cues and spoken feedback assisted me in identifying and rectifying errors better.	1 (3.33%)	0	3 (10%)	7 (23.33%)	19 (63.33%)
(8) I believe that teacher’s multimodal corrective feedback should be an integral part of teaching.	1 (3.33%)	1 (3.33%)	2 (6.67%)	1 (3.33%)	25 (83.33%)
(9) I prefer receiving multimodal corrective feedback from the teacher over written corrective feedback.	0	1 (3.33%)	2 (6.67%)	4 (13.33%)	23 (76.67%)

## 4. Methodology

### 4.1. Research design

This research aims to deepen the understanding of the perceived distinctions between various modalities that evoke emotions and to provide valuable insights for educators and practitioners regarding the potential implications of modality selection. These insights will, in turn, aid in the development of more effective setups for foreign language teaching. The specific focus of this study is on the context of German as a Foreign Language (GFL) academic writing classes. It is motivated by the observation that, compared to English learning, German learning often requires a higher degree of emotional support from teachers. This heightened need for emotional support can be attributed to factors such as the grammatical complexity of the German language, structural differences from learners’ native languages, and the presence of cultural barriers.

Conducting a multimodal affective analysis of CF in foreign language education can potentially yield deeper insights into how different modes exert their influence on student emotions. Particularly, the emotion triggered in response to teachers’ MCF holds the promise of enhancing students’ learning experiences, leading to improved comprehension. The multimodal affective approach enables effective comparisons between different modalities of emotional expression. Researchers can assess the relative impact of verbal and nonverbal elements in delivering CF, shedding light on which aspects are more influential in evoking specific emotional responses. This comparative analysis can guide instructional practices.

Nevertheless, a challenge persists in terms of developing an approach that can examine both textual and video feedback within a consistent and equally relevant framework. This need for uniformity in analysis methods is crucial to facilitate meaningful comparisons of students’ emotional responses. By adopting a multimodal affective perspective in the study of emotions within the CF process, we can better address the pedagogical demands of foreign language teaching and gain a more nuanced understanding of the interplay between emotion and academic literacy development. This paper aims to answer the following questions:

1. How do different modalities of corrective feedback (e.g., WCF, MCF) influence the intellectual engagement and emotional responses of GFL learners?
2. What pedagogical implications can be derived from the study’s findings to optimize the use of MCF in GFL academic writing classes?

### 4.2. Participants

To shed light on emotions’ real impacts on GFL, we conduct an empirical study within the context of a Chinese university, involving a cohort of 30 students who were actively enrolled in an academic German academic writing class. Participants were selected to ensure a balanced representation of gender and language proficiency levels. They were assured that their participation was entirely voluntary and that they possessed the autonomy to withdraw their consent or discontinue their involvement at any point during the research process without any repercussions.

### 4.3. Procedure

The empirical phase of this study spanned 8 weeks within the context of a Chinese university. This empirical inquiry was structured into two distinct phases, each lasting 4 weeks. In the initial phase, a cohort of 30 students underwent language learning sessions during which they were exclusively exposed to written corrective feedback (WCF) provided by their instructors. Subsequently, in the second phase, the same group of participants experienced a transition to a multimodal corrective feedback (MCF) format, delivered through videos.

Throughout both phases, we closely observed and documented the emotional experiences of the learners during their interactions with corrective feedback. To facilitate this analysis, we applied our multimodal affective approach, which allows for a comprehensive exploration of emotional responses within the context of foreign language learning.

### 4.4. Instruments

To collect data, two research instruments were deployed: a 10-item questionnaire and an in-depth interview. The questionnaire operation employed a 5-point Likert Scale encompassing categories of strongly agree, agree, neutral, disagree, and strongly disagree. Additionally, the questionnaire encompassed open-ended components that let students articulate their perceptions concerning both teacher-provided multimodal and written corrective feedback. During these interviews, participants engaged in discussions related to their emotional states concerning the teacher’s corrective feedback. This approach offers insights into the intricate interplay of multiple modes within teachers’ CF, considering both their application in the classroom and the emotional atmosphere that arises as a result.

### 4.5. Data analysis

This study used quantitative and qualitative methods to analyze the data gathered from student responses and interviews. The integration of both quantitative and qualitative analyses provided a comprehensive view of the data.

1. Quantitative analysis: To assess students’ preferences regarding corrective feedback modalities, we used descriptive statistics. Specifically, we calculated the percentage of students who strongly agreed or agreed with the statements related to MCF and WCF. These percentages provided a clear quantification of student preferences.
2. Qualitative analysis: In addition to quantitative analysis, we conducted a qualitative analysis of the interview data. We employed thematic analysis to identify recurring themes and patterns in the participants’ responses.

## 5. Results

The results yielded by this investigation shed light on the concept that the choice of corrective feedback modality during feedback delivery could influence students’ emotional states. The methods employed in this study have indicated the promise of the multimodal affective framework as an effective analytical instrument. By understanding the mediating function of emotion in academic literacy development, GFL teachers can adopt a practical approach to handling students’ emotional difficulties. This research provides a template that can be applied to a variety of scenarios.

The first goal of this study was to examine students’ preferences regarding teacher-provided corrective feedback. The findings revealed that a majority of students perceive MCF as pivotal to enhancing their English language skills, as evidenced by their responses. Among the participants, 23 students (77%) strongly agreed and 3 students (10%) agreed.

The impact of the teacher’s MCF was multifaceted. It was found that such feedback could effectively engage learners’ attention. As illuminated by participants during the interviews, when addressing complex aspects like conditional sentence formulation, learners faced difficulties. Nonetheless, the teacher’s MCF combined with hand gestures, gaze, and body language assisted her in overcoming this challenge. This observation confirms the significance of employing multimodal strategies in pedagogical interactions. The amalgamation of various modes of communication not only captured learners’ attention but also provided a comprehensive and holistic learning experience.

It is noteworthy that participants also acknowledge the role of teacher-provided MCF as a positive factor contributing to enjoyment, with 22 students (73%) strongly agreed and 4 students (13%) agreed. For comparison, students’ perception of WCF was mainly marked a neutral experience by 14 out of 30 participants (47%). As a showcase, one participant aptly articulated this experience stating “Having studied foreign language studies for so many years, I find myself emotionally detached. I have become used to encountering errors and consequently correcting them.” The teacher’s MCF in this study can be regarded as feedback imbued with positive emotions due to three factors:

1. The teacher’s skillful use of semiotic resources effectively managed the challenge of learners committing errors in the class, with 13 participants strongly agreed and 7 students (23%) agreed. For instance, when a learner made a mistake, the teacher’s sustained eye contact accompanied by a smile served to motivate them to approach this challenge with enthusiasm.
2. The teacher’s MCF effectively heightened learners’ attention. For example, when the teacher altered his intonation while pointing out an error, this action redirected the learner’s focus toward the correct form.
3. The teacher’s nuanced multimodal approach facilitated a heightened intellectual engagement with the learners’ focus on the correct form. By using specific gestures to point out errors, the teacher guided the learners’ understanding of the correct forms.

Overall, over 90% of students (4 agreed and 23 strongly agreed) emphasized the significant role of the teacher’s multimodal approach in delivering CF, attributing a sense of enjoyment and heightened attention to these interactions. Similarly, nearly all interviewed students were in agreement that the teacher’s use of multimodal feedback served as an enjoyable method of increasing their awareness of errors and their appropriate forms.

Nonverbal resources, such as eye gaze and gestures, have proven to be more potent than mere written commands in providing corrective feedback. They foster a sense of involvement and bridge the gap between teachers and students. The findings underscore the significance of teachers employing affective resources to convey emotions and assess stances that can potentially influence students’ emotional states. The teacher’s multimodal feedback assists in offering a more accurate interpretation of the intended purpose. Through understanding a wide range of verbal and nonverbal affective factors, students can enhance their ability to grasp information more effectively. For instance, when explaining a complex grammatical structure in the subordinate clause of the German language, the teacher might use their arms to visually represent the relationships between different elements of the sentence. They might use specific arm movements to depict subjects, objects, verbs, and other grammatical components. These gestures can help create a visual representation of the sentence’s structure, making it more comprehensible to the learners.

The teacher’s multimodal correction is effective due to the interplay between verbal and nonverbal cues. At times, these nonverbal cues align with verbal signals, conveying the same meanings. Additionally, there are situations where the teacher’s nonverbal cues create a clearer structure for learners to comprehend the corrective message. For example, the teacher’s posture helps learners understand the corrective intention of the written forms.

## 6. Discussion

The above empirical study shows that the expression and identification of emotions by individuals are recognized to be multifaceted. The implementation of a multimodal affective perspective offers the potential to augment our knowledge of the interplay between academic functioning and emotions, as it enables chances for new research directions. We discuss our emotional study results’ implications and the potential future work.

### 6.1. The pedagogy approach of the multimodal affective approach in academic writing

The findings of this study confirm the significance of multimodal corrective feedback in academic writing. Through the nuanced selection of modes and the integration of various cues, educators can possess the means to enhance learners’ engagement, comprehension, and enjoyment (Liu, 2022). For example, non-verbal cues like eye gaze or gestures may be more useful than written text when conveying instructions to students. MCF offers more support for foreign language acquisition. The MCF not only accommodates diverse comments for students’ written production but also presents content that resonates with learners on a personal level in a multimodal manner. Through attentive consideration of instructors’ visual and verbal cues, learners exhibit a heightened ability to discern corrective cues. The findings underscore the effectiveness and interest of the teachers’ CF with an amalgamation of hand gestures, gaze, silence, and shifts in intonation.

Our research reinforces the notion put forth by Sansuán (2020) regarding the intricate relationship between emotions and literacy education. Our study extends this perspective by illustrating that multimodal teaching methods can serve as a means for instructors to regulate students’ attitudes. The incorporation of the multimodal affective approach in the realm of academic writing manifests a multifaceted paradigm for educational instruction. By encompassing both verbal and non-verbal elements, educators can foster an enriched learning environment that is more attuned to students’ cognitive and emotional nuances (Unsworth and Mills, 2020). This holistic approach recognizes that education is not merely about the transmission of knowledge but also about nurturing the emotional well-being of learners. While prior research has explored multimodality in language learning (Jewitt, 2013), the focus on the affective dimension in this study prompts further investigations into how emotions can be intentionally harnessed to improve language education.

Our finding aligns with the work of Han and Hyland (2019), who emphasized the importance of academic emotions in written corrective feedback situations. Their contention that positive emotions, such as enjoyment and engagement, are pivotal for effective learning resonates with our findings. Our study further demonstrates that multimodal approaches contribute to creating a positive emotional ambiance that broadens learners’ attention and engagement. Moreover, our results indicate that when students become emotionally engaged through diverse modalities, they are inclined to display heightened levels of engagement.

Taking into account the above verbal and nonverbal affective dimensions of corrective feedback, this study demonstrates the potential of a teacher’s multimodal corrective feedback to foster enjoyment in a GFL writing course. The findings confirm the inherent multimodality of corrective feedback and emotion. These dimensions encompassed students’ heightened attention, intensified focus on the corrected structures, and an improved understanding of the teacher’s corrective discourse. From a pedagogical standpoint, the outcomes of this case study highlight the importance of increasing teachers’ awareness regarding the multimodal nature of their corrective feedback and its role in creating a positive emotional ambiance. They counteract the presumed link of learners’ negative emotional reactions to corrective feedback and instead cultivate a more positive atmosphere that broadens learners’ attention and engagement.

The analysis illustrates discrepancies delivered across two modalities of CF, even when originating from the same instructor. This result can guide instructors into the potential influence of technology selection on feedback modalities. It can empower educators to align technological tools with pedagogical objectives, thereby optimizing their instructional strategies. Educators may utilize multimodal teaching methods to match students’ needs, which could aid in the enhancement of their emotion regulation. During the instructional process, teachers may integrate non-verbal resources such as images, body language, and other materials to communicate their attitudes more clearly, which may be more accessible and comprehensible for students learning foreign languages. The implications of this study extend beyond the realm of German academic writing and foreign language teaching. They resonate with the broader field of education, emphasizing the importance of recognizing and harnessing the affective dimension of learning.

### 6.2. Multimodal affective approach in assessing students’ emotions

Our proposed multimodal affective approach can also assist in assessing students’ emotional responses. The realization that different modes of feedback can elicit disparate emotional reactions highlights the intricate relationship between pedagogical methods and students’ affective states. By emphasizing students’ enjoyment and building their confidence, teachers may extend their multimodal expression of attitude by utilizing different choices of writing feedback, as writing suggestions could be expressed with diverse types. The study suggests teachers could align the mode of expression with their specific pedagogical intents.

This study’s focus primarily lies in exploring students’ emotions through self-report measures, taking into account the interplay of multimodal elements in eliciting these emotions. However, as we delve deeper into the domain of MCF and its implications for foreign language learning, a more comprehensive approach could involve a meticulous multimodal analysis of students’ emotional reactions (Liu, 2022).

Such a comprehensive investigation could encompass various modalities, including verbal and non-verbal cues, that contribute to the holistic emotional experience within the context of foreign language learning. By embracing this approach, we could gain a better understanding of the nuanced ways in which different modes of communication interact with one another and jointly influence students’ affective engagement.

### 6.3. Future work

It is important to acknowledge certain limitations inherent in the application of the multimodal affective approach that deserves future work. To begin with, the successful implementation of this approach relies on educators’ proficiency in navigating diverse modalities of teaching, which may necessitate additional training and workload for them. Our study offers valuable insights for educators to select the appropriate modalities and stimulate students’ positive emotions in the context of GFL and can be extendable to other language learning contexts in future work. Future research could also focus on designing effective training programs that equip educators with the skills to navigate diverse modalities efficiently.

The current study focuses on delving into the emotional dimensions of corrective feedback. It is worth extensively delving into the broader contextual factors that might influence students’ emotional responses, which we leave as a future work. Emotion within academic literacy development should be understood as an interface between individual and their environment, which is mediated by social contexts (Mulligan and Scherer, 2012). For instance, delving into individual differences such as cultural backgrounds, learning preferences, and personality traits might unveil additional layers of complexity in the emotional interplay. This extended perspective could also involve the exploration of potential mediators that might either enhance or mitigate the impact of certain multimodal cues on emotional states.

Furthermore, students’ emotions can be assessed through heterogeneous sources to obtain a comprehensive understanding of emotion and academic literacy development. Emerging technologies, such as wearable devices for real-time emotion tracking and advanced neuroimaging techniques, could shed further light on the neural and physiological underpinnings of emotional responses during educational interactions. For example, emotional triggers can cause a student’s heart rate to change, their facial expression to alter, and their muscles to tense. The integration of deep-learning-based fusion methods for analyzing multimodal data from heterogeneous sources (Huang et al., 2020), presents a promising trajectory. Models like Word2Vec (Mikolov et al., 2013), GloVe (Sakketou and Ampazis, 2020), Transformer network (Huang et al., 2020) and BERT (Su et al., 2019) could be leveraged to enhance the understanding of different modalities in teaching.

Lastly, the implementation of a multimodal affective method, adept at detecting, analyzing, and interpreting expressions across various modes corresponding to diverse emotional states, can enrich pedagogical practices. Through a better understanding of emotional knowledge, educators may optimize their teaching methods by employing various modalities to alleviate student learning stress and enhance their learning interests (Wang and Ye, 2021). To address these issues, future investigations can enhance the application of the multimodal affective approach in language education and contribute to the creation of a more supportive and emotionally responsive learning environment.

## 7. Conclusion

This investigation advances the proposition that emotion constitutes a complex, multi-faceted construct, advocating for the adoption of a multi-dimensional approach to dissecting and comprehending emotion. The present study introduces a multimodal affective approach for dissecting emotion, offering a versatile tool applicable across diverse scenarios, such as within the realm of teacher-student interactions, particularly evident in the context of the provision of corrective feedback.

This finding highlights the significance of incorporating multimodal emotion in the GFL writing curriculum and underscores the need for multimodal teaching strategies. Through an in-depth analysis, we have uncovered the potential of integrating diverse modalities of expression in the educational process. The study’s findings underscore the importance of considering not only verbal but also non-verbal elements when designing teaching strategies. Multimodal approaches provide educators with versatile tools to create an enriched learning environment that caters to student’s cognitive and emotional nuances. From incorporating gestures to utilizing images and body language, educators can effectively enhance their ability to convey complex concepts and emotions.

Furthermore, this research introduces a multimodal affective perspective that has broader applicability in education. It offers new insights into the intricate relationship between academic literacy development and emotions, encouraging foreign language teachers to regulate students’ emotions through a multimodal approach. Despite the numerous benefits of multimodality, it is important to acknowledge that its successful implementation may require additional training for educators. Future work should focus on designing effective training programs that equip teachers with the skills needed to navigate diverse modalities efficiently.

In a rapidly evolving educational landscape, the study of multimodality in language education opens up exciting avenues for research and pedagogical innovation. As technology advances and our understanding of emotional engagement deepens, multimodal approaches are poised to play a pivotal role in creating supportive and emotionally responsive learning environments.

## Data availability statement

The raw data supporting the conclusions of this article will be made available by the authors, without undue reservation.

## Author contributions

XG: Writing – original draft, Writing – review & editing.
